# Head and neck dermatofibrosarcoma protuberans: Case series of extensive resections and reconstructions with literature review

**DOI:** 10.12688/f1000research.162699.1

**Published:** 2025-03-14

**Authors:** Skander Kedous, Ameni Amri, Alia Methnani, Yasmine Fertani, Amira Gallas, Rim Braham, Mohamed Dhaha, Souhail Jbali, Sawssen Dhembri

**Affiliations:** 1 ENT Head & Neck Surgery Department, Salah Azaiz Institute, Tunis, Tunisia; 2 Plastic and Reconstructive Surgery Department, Salah Azaiz Institute, Tunis, Tunisia

**Keywords:** adjuvant radiotherapy, dermatofibrosarcoma, free flap, head and neck, reconstruction

## Abstract

Dermatofibrosarcoma protuberans is a rare, locally aggressive soft-tissue sarcoma. Head and neck involvement accounts for only 10–15% of cases. Achieving clear margins in this region is challenging owing to anatomical constraints. Such cases often require extensive resection and complex reconstructions. This study presents a case series of extensive dermatofibrosarcoma resections in the head and neck, focusing on the surgical margins, reconstruction strategies, recurrence rates, and adjuvant therapy. We report four cases of head and neck dermatofibrosarcoma involving the cheek and scalp. Surgery included wide local excision with margins of 3–5 cm, which was confirmed intraoperatively by frozen section analysis. Reconstruction involved free flaps, local flaps, and healing with secondary intention. The choice depends on the defect size and location. Adjuvant radiotherapy was administered to selected cases. All patients achieved negative margins. One patient developed flap necrosis that required revision surgery. No local recurrence was observed during the follow-up (1–7 years). Head and neck dermatofibrosarcoma justifies aggressive surgical resection to achieve clear margins, which is the key to reducing the risk of recurrence. Free flaps and local reconstruction techniques ensure good functional and aesthetic outcomes. Adjuvant radiotherapy is indicated in patients with close margins or deep invasion. Long-term follow-up is essential owing to its high recurrence potential.

## Introduction

Dermatofibrosarcoma protuberans (DFSP) is a rare aggressive soft tissue sarcoma. It arises from the dermis and slowly invades the subcutaneous tissues. DFSP often exhibits high recurrence rates owing to its infiltrative nature. Head and neck involvement is uncommon, accounting for 10–15% of cases. ^1, 2^ These regions present surgical challenges owing to their functional and aesthetic obligations. The key treatment is wide local excision with margins of at least 2–3 cm. ^3^ Reconstructive approaches vary based on the defect size and location. Options range from local flaps to free tissue transfer. ^4^ In addition to presenting a case series of four patients with head and neck DFSP, we reviewed the literature to highlight the current evidence on surgical management, reconstructive strategies, recurrence risk, and the role of adjuvant therapy.

## Case reports

### Case 1

**Figure 1.**
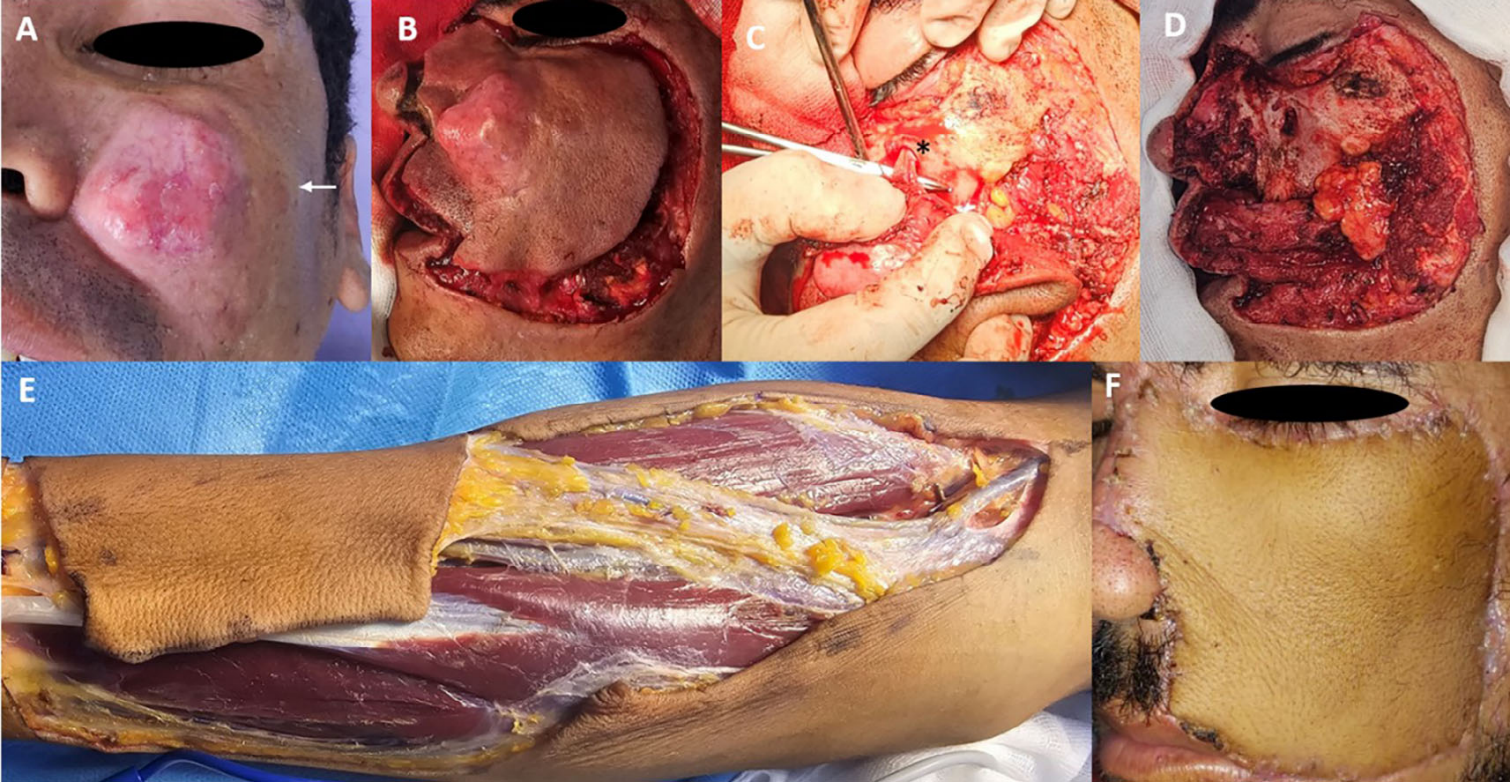
Five centimeters dermo-hypodermic mass with poorly defined borders and a 1 cm satellite lesion in the zygomatic area (white arrow) (A). Primary definition of operating margins (B). Identification of infraorbital nerve and infraorbital canal (black asterisk) (C). Final defect after tumor removal (D) Right forearm flap harvesting (E). Flap appearance at 40 days postoperatively (F).

A 32-year-old male presented with a progressively enlarging painless nodule on his left cheek over the past year. The patient had no significant medical history. Clinical examination revealed a firm, dermo-hypodermic mass measuring approximately 5 cm, with poorly defined borders and a 1 cm satellite lesion in the zygomatic area ( Figure 1A). There were no overlying buccal mucosal abnormalities or cervical lymphadenopathies. MRI revealed a subcutaneous left jugal tumor (30 × 24 × 30 mm) extending to the anterior wall of the maxillary sinus without clear signs of invasion. A biopsy confirmed DFSP, which was supported by CD34 positivity. The patient underwent tumor excision with 1-3 cm margins, which was confirmed intraoperatively by frozen section analysis. The resection margins extended deeply to the zygomatic bone, maxilla, and masseter muscle, medially to the nasal bones and left alar cartilage, which was resected with 1 cm margins ( Figure 1B, 1D). The infraorbital nerve was resected, with a frozen section margin assessment at the level of its entrance into the infraorbital foramen, which was found to be healthy ( Figure 1C). The defect was reconstructed using a radial forearm free flap ( Figure 1E). Vascular anastomosis was performed between the radial artery and the facial artery and between the cephalic vein and the facial vein. The reconstruction of the alar subunit was performed as the second step. Histopathology confirmed negative surgical margins, with a deep margin of 5 mm and no evidence of perineural or vascular invasion. Owing to the tumor size and proximity to deep structures, the multidisciplinary team recommended adjuvant radiotherapy to further minimize the risk of recurrence. The patient remained disease-free after eight months of follow-up ( Figure 1F).

### Case 2

**Figure 2.**
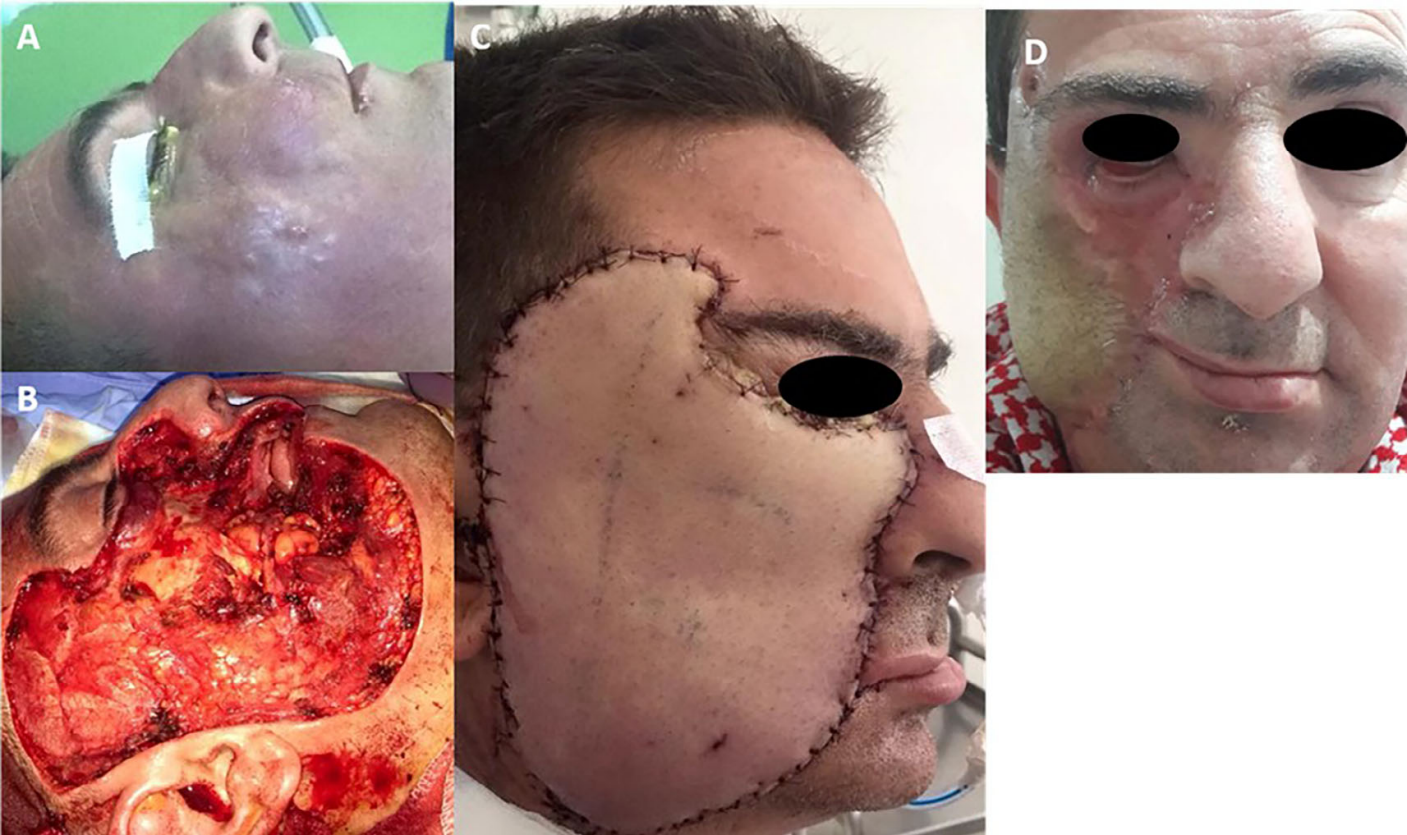
Six centimeters poorly defined mass of the right cheek (A). Large facial defect of soft tissue (B). Result of the reconstruction with antero-lateral thigh flap five days after second surgery (C). Retraction of the flap after radiation, photography taken at 6 months postoperatively (D).

A 40-year-old male patient presented with a rapidly enlarging subcutaneous mass in the right cheek. Clinical examination revealed a firm right jugal mass extending towards the right zygomatic region and infraorbital tissues ( Figure 2A). There was no orbital or intraoral involvement. Biopsy confirmed DFSP. Magnetic resonance imaging (MRI) showed a 6 cm poorly defined soft tissue mass, highly vascularized, infiltrating the buccinator, orbicularis oris, zygomaticus muscles, and nasogenian region. Resection involved the right infraorbital and lower palpebral regions, with margins extending to the right oral mucosa ( Figure 2B). A free anterolateral thigh flap was harvested for defect coverage, with microvascular anastomosis to the facial pedicle ( Figure 2C). Histopathological analysis confirmed the diagnosis of DFSP. The resection margins were clear, with a lateral safety margin of 4 mm and deep margin of 1 mm. Postoperatively, the patient developed partial flap necrosis and vascular pedicle thrombosis. During the revision surgery, the inferior thyroid artery was used as the recipient vessel for microvascular anastomosis. Adjuvant radiotherapy (54 Gy) was administered postoperatively, given close margins. The patient experienced ectropion of the right lower eyelid and retraction of the right labial commissure, secondary to flap contraction ( Figure 2D). The patient underwent corrective surgery for ectropion and Z-plasty of the oral commissure. A follow-up MRI showed no evidence of recurrence. The patient has remained disease-free for six years.

### Case 3

**Figure 3.**
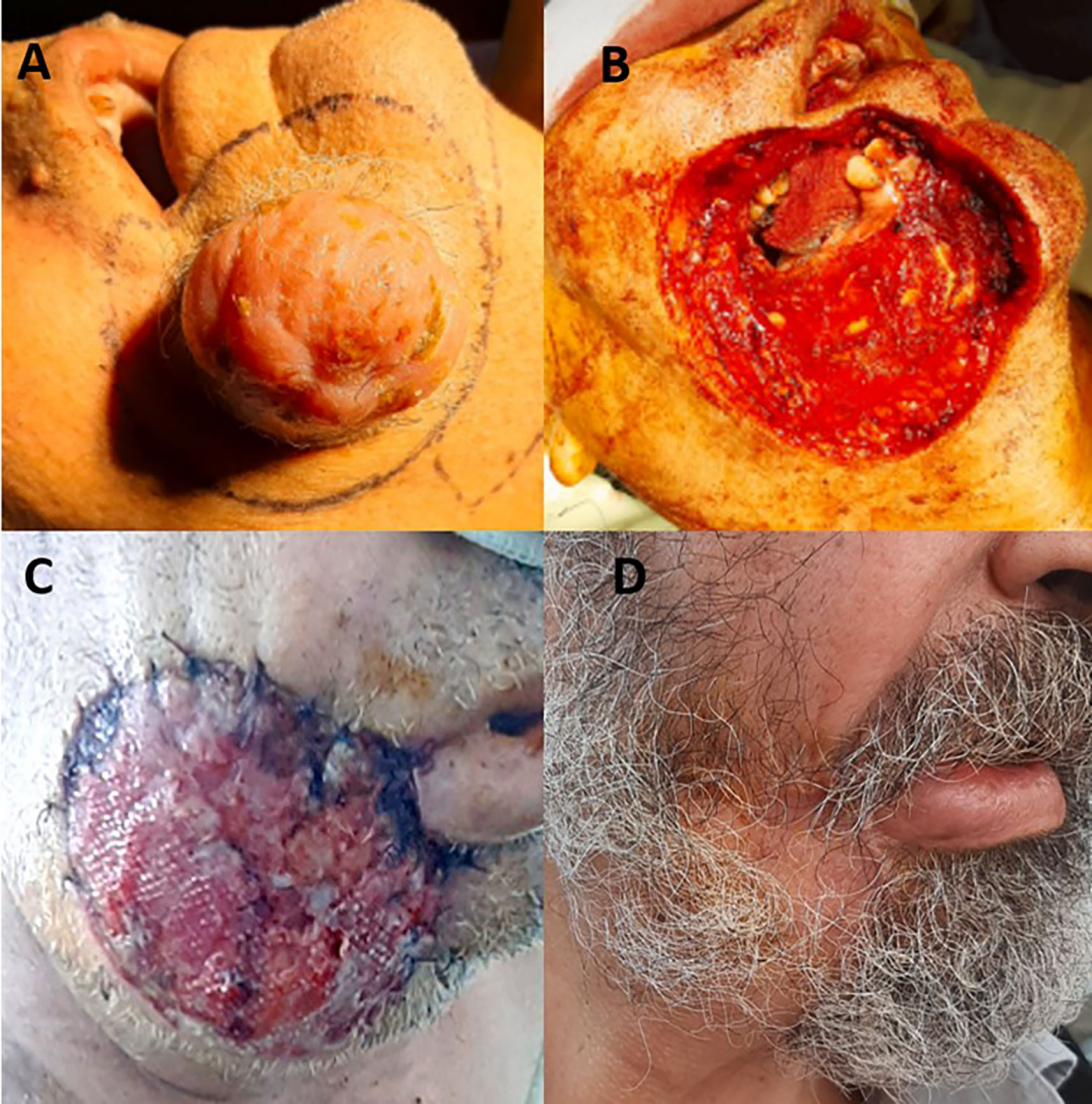
Intraoperative appearance of the tumor before surgical excision (A). Resulting skin and soft tissue defect including underlying mucosa (B). Appearance of pectoralis major flap with the muscular surface on the outside (C). Epithelization of the muscular surface 10 months postoperatively (D).

A 68-year-old man with no significant medical history presented with a one-year history of progressive enlargement and painless swelling of the right cheek. Clinical examination revealed a firm, mobile, non-ulcerated mass, measuring 4 cm in diameter. Mucosal or bony involvement was not observed. The overlying skin exhibited mild inflammation. Imaging confirmed a subcutaneous mass opposite the right mandibular ramus, without bony invasion. No signs of distant metastasis were observed. Magnetic resonance imaging (MRI) revealed a well-defined lesion with heterogeneous enhancement. Incisional biopsy revealed proliferation of spindle-shaped cells arranged in a “woven basket” pattern, confirming DFSP. Immunohistochemistry results were positive for CD34. The patient underwent tumor excision with wide margins of 2-3 cm including the underlying oral mucosa ( Figure 3A, B). Perioperative frozen sections of the margins were clear. Defect reconstruction was performed by using a pectoralis major myocutaneous flap. The skin covered the mucosal defect, whereas the muscular side provided coverage of the outer surface ( Figure 3C). The postoperative course was uneventful. The outer surface of the flap epithelialized over time ( Figure 3D). Histopathology confirmed the diagnosis, showing the proliferation of spindle cells with mild atypia. The tumor margins were tumor-free. Radiotherapy was not administered in this case. No recurrence was observed during the one-year follow-up period.

### Case 4

**Figure 4.**
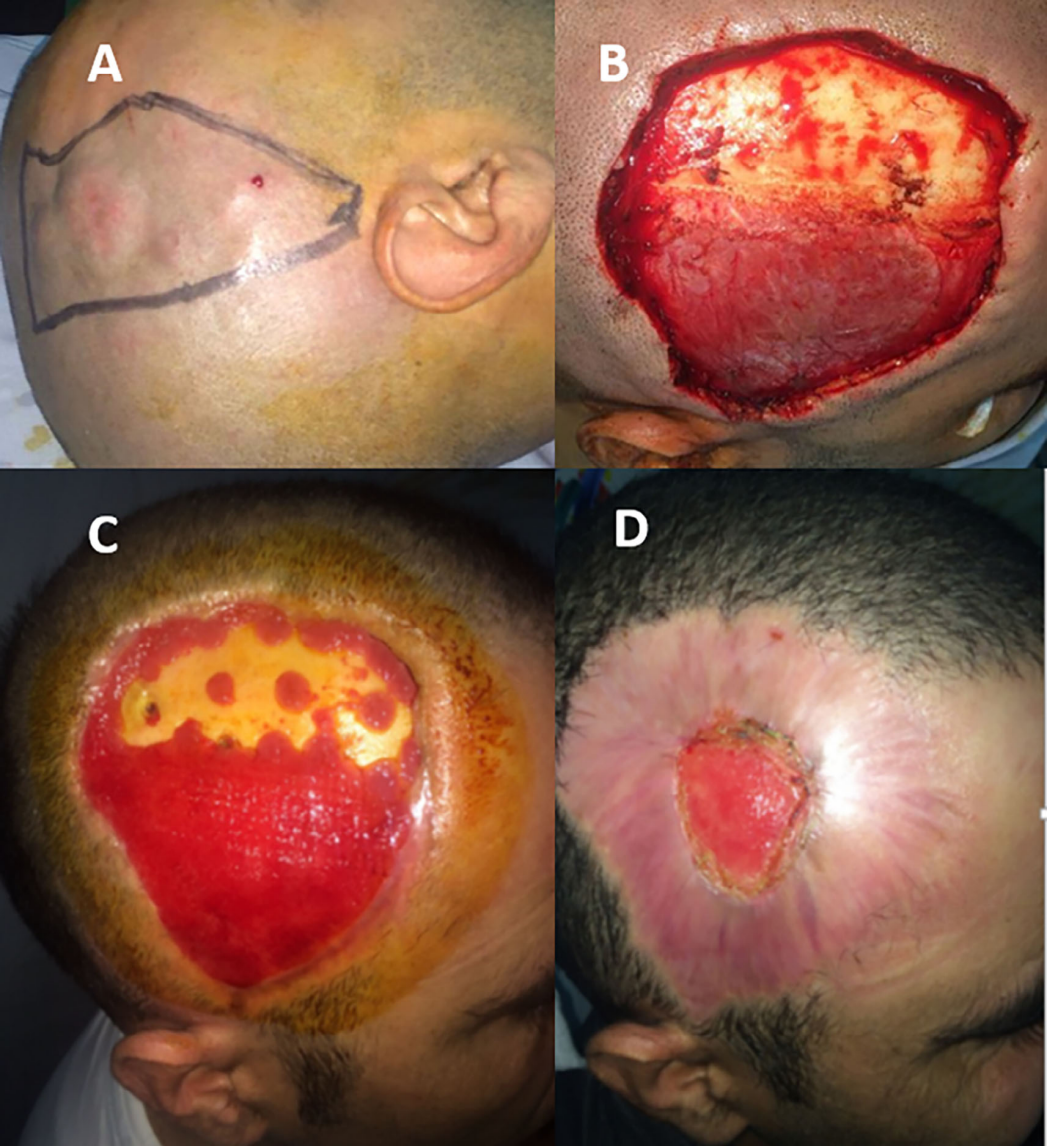
Main tumor surrounded by several satellite lesions, extending from the right frontal to the temporal and parietal regions (A). Scalp defect including the pericranium (B). Progression of the secondary intension healing process at 15 (C) and 60 days (D).

A 33-year-old male presented with a firm, slow-growing, and non-tender scalp mass that had evolved over several years. Clinical examination revealed a poorly defined, indurated tumor formed by a 3.5 cm main lesion surrounded by several satellite lesions extending from the right frontal to the temporal and parietal regions, reaching 2 cm above the insertion of the helix ( Figure 4A). CT and MRI revealed a lobulated subcutaneous mass measuring 6 cm × 3 cm. Bone invasion and intracranial extension were not observed. The patient underwent wide local excision with 3-4 cm margins, including the pericranium, resulting in a final defect extending from the frontal to parieto-occipital region ( Figure 4B). Multiple frozen section analyses confirmed the presence of clear margins. The surgical defect healed by secondary intention, and a thin skin graft was not necessary in this particular case ( Figure 4C, D). The pathology report confirmed the diagnosis, with clear margins, particularly the deep ones. Adjuvant radiotherapy was not administered. The patient was offered secondary skin expansion of the scalp to guarantee capillary coverage; however, he refused. The patient remained recurrence-free at the 7-year follow-up.

## Discussion

DFSP is a rare soft-tissue sarcoma. Although slow-growing, this malignancy is locally aggressive. It accounts for approximately 1% of all soft tissue sarcomas. Head and neck involvement is rare, accounting for only 10-15% of cases. In contrast to its exceptional metastatic potential, DFSP is characterized by a high tendency for local recurrence. This risk underscores the importance of achieving adequate surgical margins. ^5^ The recurrence rate depends widely on the anatomical location and type of treatment for these tumors. In fact, involvement of the head and neck is associated with a recurrence rate of 30-50%. This rate is particularly alarming, as it is higher than those observed in other anatomical regions. Furthermore, this risk increases in cases with deep local extension, such as involvement of the periosteum, muscle, or bone. ^6^ When it comes to molecular and genetic characteristics, DFSP is characterized by the COL1A1-PDGFB fusion gene. This alteration results in the constitutive activation of the PDGFRB pathway. This phenomenon is the direct mechanism underlying uncontrolled fibroblast proliferation. ^6^ The identification of this particular genetic alteration is a key diagnostic marker for managing these cases. This has led to the development of targeted therapies, such as imatinib. Targeted therapies have revolutionized the management of unresectable, recurrent, or metastatic DFSP by targeting PDGFRB. In fact, tumor regression under imatinib has been documented to be up to 70%, allowing delayed surgical excision in some cases. However, discontinuation of imatinib may lead to recurrence, which is why long-term follow-up is crucial. ^2^^,^^7^ DFSP with fibrosarcomatous transformation (DFSP-FS) is a challenging procedure. This variant is associated with higher mitotic activity, increased cellular atypia, and a major risk of distant metastasis than conventional DFSP. This variant accounts for approximately 10-15% of all DFSP cases. The reported metastatic rate is as high as 16%. ^8^ Distant secondary lesions commonly affect the lungs. ^2, 9, 10^ Regarding clinical presentation, DFSP of the head and neck, particularly in the early stages, can be misdiagnosed as a benign lesion. Typically, the tumor presents as a slow-growing, painless mass on the skin. Over several months or even years, the lesion turned into firm, reddish-brown, or violaceous nodules or plaques. Along with their slow-growing character, DFSP lesions can enter fast-growing phases, stimulating patients to consult. ^4, 5^ Facial and scalp involvement was associated with an infiltrative extension pattern. This feature increases the potential for invasion into deep structures, such as the periosteum, muscle, or even bone, which increases the risk of recurrence. ^11^ Another recurrence factor was dimension. Large tumors (>5 cm) were associated with a higher recurrence rate. The risk increases even more when the tumor involves areas in which wide surgical margins are difficult to achieve. The American Musculoskeletal Tumor Society has adopted a staging system for DSFP, which has also been adopted by the German Guidelines. Low-grade intra-compartmental lesions were classified as IA. When these low-grade tumors exhibit extra-compartmental extension into the underlying fascia or muscle, they are staged as IB. ^12, 13^ In establishing the diagnosis of DFSP, evaluation relies on a combination of histopathology, immunohistochemistry, and imaging studies. Histologically, DFSP exhibits a typical storiform or whorled pattern of spindle cells invading the dermis and subcutaneous tissue. The tumor cells were CD34-positive and negative for desmin, SMA, and S100. These features are key to distinguishing DFSP from other spindle cell neoplasms. ^3, 14^ Regarding imaging, a study on MRI findings in DFSP revealed that T2 hyperintensity and marked enhancement are almost universal features in these lesions. ^15^ Ophthalmologic DFSP is a rare entity with a limited number of reported cases. A systematic review of the literature on the ophthalmologic aspects of DFSP highlights its rarity in the orbital and periorbital regions, with only a few reported cases. DFSP in this location often presents as a painless firm mass, sometimes causing proptosis, diplopia, or nasolacrimal duct obstruction. Most cases are treated with wide local excision or enbloc resection, aiming for clear margins while preserving ocular function. In more advanced cases with orbital invasion, orbital exenteration is performed, sometimes combined with dacryocystectomy for lacrimal sac involvement. Mohs micrographic surgery has occasionally been utilized to minimize the risk of recurrence. ^16^ For cases with close or positive margins and when further surgical resection is not feasible, adjuvant radiotherapy with IMRT has been suggested. Even after negative-margin resection, periorbital DFSP has shown recurrence. This highlights the importance of a long-term follow-up. ^17^ Due to the thin nature of soft tissues and their proximity to the skull, scalp involvement is challenging. The recurrence rate for scalp DFSP was significantly higher than that for other anatomical locations. This rate is reported to be 50-75%. Unlike the trunk, scalp DFSP often justifies aggressive wide resection. In such cases, treatment includes periosteal and bony excision. A literature review of 74 scalp DFSP cases with skull involvement revealed that periosteal invasion was reported in nearly all cases that required wide excision. Skull invasion was documented in five cases, requiring margins of at least 20 mm into the bone to achieve local control. However, intracranial extension was an exception, with only 17 reported cases. Most intracranial involvements are due to inadequate initial resection. When tumor invasion is limited to the periosteum, literature suggests that dermal resection with periosteal removal is an effective strategy for local control. However, if skull involvement is confirmed, marginal bone resection at least 20 mm from the tumor edge is recommended to ensure negative margins. ^18^ Another exceedingly rare location is the oral DFSP, accounting for less than 5% of all head and neck cases. The most affected area is the buccal mucosa; however, cases have also been reported in the lip and gingiva. Owing to the limited tissue availability and functional constraints, it is challenging to achieve clear surgical margins in the oral cavity. Martinez et al. reported a case of DFSP of the buccal mucosa where initial margins were positive after an initial wide excision, requiring an additional 20 mm re-excision. A review of five intraoral DFSP cases reported that excision margins varied from 15 to 20 mm, with no documented recurrences. However, the long-term follow-up findings remain limited. Reconstruction in oral cases can be performed by using either local or free flaps. The Chinese flap is a common option because of its pliability and vascularity. ^19^ In other locations, lesions exceeding 10 cm in diameter have been associated with major recurrence rates, particularly when located on the scalp or face. Large DFSP lesions exhibit an aggressive behavior. These can be associated with deep tissue invasion, ulceration, and increased risk of metastasis. Therefore, to minimize the risk of recurrence, wide local excision with margins > 2 cm is recommended for large, high-risk tumors. When skull involvement is confirmed, periosteal resection and potential craniectomy are crucial. ^10^ For aggressive extensions, when surgery is not feasible, or in cases of residual microscopic disease, adjuvant therapy, including radiotherapy and targeted therapy (imatinib), may be considered. The gold standard treatment for DFSP, particularly in the head and neck, requires wide local excision with 2–5 cm margins to reduce the risk of recurrence. ^20^ Established recurrence risk factors are dominated by margins <2 cm, as they are associated with recurrence rates of up to 60%. ^5^ In contrast, surgical margins >3 cm reduce recurrence rates to < 10%. ^20^ Hence, when the pathology confirms positive or close (<1 mm) margins, re-excision is strongly recommended. When it comes to periosteal and deep margin considerations, frozen section analysis can be used intraoperatively to assess the margin clearance. However, its reliability is debatable because of DFSP’s infiltrative growth pattern of DFSP. For challenging scalp DFSP cases, slow Mohs surgery is an effective alternative to preserve healthy tissue while ensuring oncologic control. ^21^ The aim of Mohs micrographic surgery is to reduce the recurrence rates. This technique facilitates precise excision of DFSP by enabling comprehensive margin evaluation through enface sectioning. A study by Tom et al. demonstrated that Mohs surgery, when combined with inverted horizontal paraffin sectioning, improves tumor clearance compared to frozen-section techniques, as residual tumor was identified in 78% of cases despite initial frozen-section negativity. ^22^ Radiotherapy is an option reserved for cases where further surgery is not feasible or when fibrosarcomatous transformation is identified, thereby increasing the risk of recurrence. ^23^ Tissue expansion is frequently used in scalp reconstructions. This technique allows for single-stage reconstruction and reduces the need for free tissue transfer. For cases in which initial defect coverage is required, post-resection expansion can be performed before final aesthetic refinement. ^24^ As for the lower face and cervical involvement, the deltopectoral and parascapular flaps were expanded preoperatively. This option facilitates soft tissue coverage while preserving function. However, large scalp defects frequently require a multistage reconstruction approach with serial expansion techniques, followed by definitive local tissue advancement. ^25^ In cases where tissue conditions are suboptimal, healing by secondary intention can be considered. This allows the formation of granulation tissue before grafting or flap reconstruction. These techniques are interesting as they ensure optimal oncologic and functional outcomes, especially when reconstruction is not feasible owing to limited vascularity for flap or graft repair. ^26^ A review of the literature highlights the anterolateral thigh flap as a preferred reconstructive option for large DFSP defects in the head and neck. ^27^ Studies emphasize its relevance when local flaps are insufficient owing to extensive tissue loss or the need for aesthetic preservation. This flap not only provides ample soft tissue coverage but also maintains functional outcomes and minimizes donor-site morbidity. Compared with the radial forearm free flap or pectoralis major flap, this flap offers better contouring with fewer complications, making it particularly valuable for full-thickness defects. Additionally, literature reviews have highlighted its role in maintaining distinct facial aesthetic subunits. Given the adaptability and reliable vascular anatomy of this flap, it remains a leading choice for the reconstruction of large DFSP excision defects. ^27, 28^ Surface mold brachytherapy is a targeted radiotherapy technique utilized in scalp cases with recurrence risk factors, or when pathological confirmation of positive or close margins and re-excision is not possible. ^23^ This technique is characterized by its precision and conformity. In fact, it delivers high-dose radiation to the tumor bed while sparing the adjacent healthy tissues. This particularity reduces toxicity compared to external beam radiation therapy. In addition, long-term local control and minimal side effects. ^23^

## Consent

Written informed consent for the publication of the case reports and of the associated images was obtained from each of the patients prior to submission.

## Data Availability

No data are associated with this article.
