# Supplementary material for: Head and neck dermatofibrosarcoma protuberans: Case series of extensive resections and reconstructions with literature review
Source: F1000Res. 2025 May 12;14:294. Originally published 2025 Mar 14. [Version 2] doi: 10.12688/f1000research.162699.2 (PMC12086507; doi:10.12688/f1000research.162699.2)
Supplement: Peer review history

# Reviewer report: Reviewer response for version 2

**DOI:** 10.5256/f1000research.181850.r384328

**Published:** 2025-05-28

**Authors:** Senda Turki

**Affiliations:** 1 Service ORL, Hôpital FSI,Faculté de Médecine de Tunis, Université Tunis El Manar, Tunis, Tunisia

the comments have been taken into account by the authors: the discussion has been separated into paragraphs making it clearer and a conclusion has been added

Are enough details provided of any physical examination and diagnostic tests, treatment given and outcomes?

Yes

Is the background of the cases’ history and progression described in sufficient detail?

Yes

Is sufficient discussion included of the importance of the findings and their relevance to future understanding of disease processes, diagnosis or treatment?

Yes

Is the conclusion balanced and justified on the basis of the findings?

Partly

Reviewer Expertise:

cervico-facial surgery, rhinology, otology, balance and vertigo, sleep apnea

I confirm that I have read this submission and believe that I have an appropriate level of expertise to confirm that it is of an acceptable scientific standard.

# Reviewer report: Reviewer response for version 1

**DOI:** 10.5256/f1000research.178941.r374041

**Published:** 2025-05-08

**Authors:** Senda Turki

**Affiliations:** 1 Service ORL, Hôpital FSI,Faculté de Médecine de Tunis, Université Tunis El Manar, Tunis, Tunisia

This article deals with a tumor which, although rare, represents a therapeutic challenge due to its localization and the high risk of recurrence.

 In fact, Dermatofibrosarcoma Protuberans poses a surgical problem when localized in the head and neck: resection, which must comply with oncological rules, coupled with suitable reconstruction, should enable the best possible preservation of the facial units for a suitable functional and aesthetic result.

**Cases presentation:**

 The authors have presented 4 very interesting cases, covering different localizations. These cases are presented in a very clear manner, with excellent iconography showing the extent of resection and the reconstruction technique, as well as the final result.

**Discussion:**

 This is a very well-conducted review, based on a rich and up-to-date bibliography. It has thus developed the main points of the management of this tumor, with particular emphasis on the importance of the risk of recurrence, particularly frequent for these localizations:

 - Genetic markers which have been the basis for the development of targeted therapy

 - Principles for the extent of resection depending on the localization, to minimize the risk of recurrence

 - Different reconstruction modalities depending on the localization and the extent of excision, well detailed

 - Importance of multidisciplinary consultation to establish the indication for adjuvant therapies, principally post-operative radiotherapy.

 However, I have two comments for the authors, which in no way detract from the quality of this article:

- it would be preferable to divide the discussion into paragraphs, to better highlight the different points of discussion
- it would be desirable to add a few concluding lines after the discussion.

Are enough details provided of any physical examination and diagnostic tests, treatment given and outcomes?

Yes

Is the background of the cases’ history and progression described in sufficient detail?

Yes

Is sufficient discussion included of the importance of the findings and their relevance to future understanding of disease processes, diagnosis or treatment?

Yes

Is the conclusion balanced and justified on the basis of the findings?

Partly

Reviewer Expertise:

cervico-facial surgery, rhinology, otology, balance and vertigo, sleep apnea

I confirm that I have read this submission and believe that I have an appropriate level of expertise to confirm that it is of an acceptable scientific standard.

# Reviewer report: Reviewer response for version 1

**DOI:** 10.5256/f1000research.178941.r374038

**Published:** 2025-04-18

**Authors:** Moncef Sellami

**Affiliations:** 1 University of Sfax, Sfax, Tunisia

This well-written and informative manuscript addresses a rare and clinically challenging presentation of dermatofibrosarcoma protuberans (DFSP) in the head and neck region.

 The authors present a case series of four patients, with detailed surgical descriptions, histopathological confirmation, and reconstructive strategies.

 The discussion is enriched by a comprehensive literature review that covers recurrence risks, genetic markers, and the role of targeted therapy.

 The emphasis on the importance of adjuvant radiotherapy in cases with close margins is appropriate. However, the decision-making process regarding when to recommend radiotherapy could be presented more clearly. Including a brief explanation of how the multidisciplinary team reached therapeutic decisions in each case would add practical value to the manuscript.

 Minor grammatical and terminological revisions are recommended throughout the manuscript.

 For example: The term “jugal” is anatomically correct but not commonly used in English-language medical literature; replacing it with “cheek” would improve clarity and align with standard clinical terminology.

Are enough details provided of any physical examination and diagnostic tests, treatment given and outcomes?

Yes

Is the background of the cases’ history and progression described in sufficient detail?

Yes

Is sufficient discussion included of the importance of the findings and their relevance to future understanding of disease processes, diagnosis or treatment?

Yes

Is the conclusion balanced and justified on the basis of the findings?

Partly

Reviewer Expertise:

otorhinolaryngology and head and neck surgery

I confirm that I have read this submission and believe that I have an appropriate level of expertise to confirm that it is of an acceptable scientific standard.
